# Spaying in Nervous Diseases

**Published:** 1887-01

**Authors:** 


SPAYING IN NERVOUS DISEASES.

A discussion on the subject of spaying or castration by Sir Spencer Wells, Dr. Alfred Hegar and Dr. Robert Battey, appears in the October number of the *American Journal of the Medical Sciences.*

Dr. Battey distinctly repudiates the doctrine that normal ovaries are to be removed for the cure of nervous affections. His paper opens as follows: "Within my knowledge, it has not been the practice of American surgeons to attempt the cure of mental and nervous disorders by the removal of healthy ovaries or of healthy tubes. The ovaries removed and the tubes as well have presented signs of disease—signs which are evident to the naked eye and palpable to the sense of touch. For the misconception on this point still existing, my own ignorance of the histology and pathology of the ovary is largely responsible, in that, during the early history of the operation, I removed ovaries which I erroneously supposed to be healthy, and gave to the operation the unfortunate and now obsolete name of 'normal ovariotomy.'" It will be seen from this that Dr. Battey does not regard the removal of normal ovaries in nervous and mental disease as ever justified.

This is substantially the position taken by Hegar, who lays down the rule as follows: "Castration is indicated in a psychosis evoked or maintained by pathological alterations in the sexual organs, and in a neurosis originating from the same source as soon as this imperils life or hinders all occupation and all enjoyment of life. * * * Castration must actually affect the cause which occasions or keeps up nervous irritation."

Sir Spencer Wells enters largely into the moral aspects of the question and assumes that healthy ovaries are being removed for the cure of nervous diseases. He considers that in nymphomania and mental diseases it is, to say the least, unjustifiable, and it should never be done without the consent of a sane patient. The operation may be advised in uterine fibroids, uncontrollable hæmorrhages, malformations and certain rare cases of ovarian dysmenorrhœa.

It will be seen that Battey and Hegar are in substantial accord on the main point at issue; regarding pathological changes in the ovaries as the possible starting point of nervous irritation, and justifying their removal in cases where this relationship can be clearly traced. It is to be regretted that Sir Spencer Wells did not discuss more fully the point at issue. We are left in some doubt as to just what his views are regarding the removal of *diseased* ovaries when a complicating factor in neuroses.

We are introduced to another aspect of this question by an interesting discussion opened by Schroeder in a recent meeting of the German Society of Naturalists (*Tagebl. d. Vers. Deutcher Naturf. N. S. W. zu Berlin*, 1886, p. 310). Schroeder adopts the division of *oöphorectomies* as given by Hegar. First, those cases in which the ovary is removed for disease of the organ, and, second, those in which they are removed to hasten the advent of the menopause. He regarded the first class as belonging entirely to general surgery, and the indications are to be arrived at by the application of general surgical principles. The ovaries are removed because they are cystically degenerated or chronically inflamed. If one ovary is diseased, it only is to be removed, and, under certain conditions, only the affected portion. The principle is, however, very different when the operation is to hasten the menopause. In these cases both ovaries must be completely removed, and the operation is at times indicated when the ovaries are healthy. This conclusion Hegar will not admit, claiming that the operation is only admissible when the effect of the menopause is desired on the genital tract (tumors, etc.), or when neuroses are dependent on some pathological change in the structure of the ovary. This view Schroeder will not acknowledge, and he forcibly observes that there is a great difference whether the operation is made for a diseased ovary, or for its effects on a tumor in the uterus. The first case does not belong to castration in its limited sense, while in the second a healthy organ is removed for its effect on another diseased organ.

In support of his views, Schroeder observes that in just the same way in general neuroses we can remove healthy ovaries to obtain a healing effect on the whole organism. Whether and how far neuroses depend on pathological changes in the ovary we know not. Hegar himself admits that they are not always found where considerable alteration has taken place.

Schroeder further observes that severe neuroses often disappear with the advent of the natural or artificial menopause without pathological changes in the ovary. Then the demand of Hegar is difficult to satisfy, as we cannot define just what is the normal condition of the ovary when small cysts or hardened condition of the stroma are to be termed pathological. The operation in uterine myomas is entirely empirical, and even so can the usefulness of the operation in neuroses be settled by experience alone.

Hegar in his reply said that he would like to correct two misunderstandings regarding his position. He had never rejected the operation for the removal of healthy ovaries, but had only insisted on the presence of an aetiological relation between the neurosis and pathological changes in the sexual apparatus. He had not absolutely rejected castration in perfectly healthy organs, but had only said that in the present state of our knowledge the operation lacked foundation. Certain it is, that severe and life-embittering nervous troubles follow sexual diseases and a cure by castration is possible. The small cystic generation and the so-called cirrhosis of the ovary are as yet but little studied affections; at times they cause little trouble, at others they are capable of causing nymphomania, epilepsy, or even insanity. It is difficult to determine the exact relation between cause and effect, but the changes in the ovary alone furnish an indication. Pregnancy, parturition, and child-bed, doubtless cause neuroses, but here other causal factors, heredity, etc., often play an important part. Much more difficult is the decision in the so-called menstrual neuroses. Hegar has never seen a case reported in which menstruation can be regarded as the sole or most important cause of a severe neurosis. Shall it then be considered that menstruation, in an intact condition of the organs, is the cause of many serious neuroses, and that its removal would heal the disease? Hegar would not acknowledge the correctness of this view. The founding of an operative indication on the effect of the climacteric on the organism is still more difficult. A quieter temperament and better nutrition are the usual results, but are they a sufficient indication for the performance of the operation? The conditions which govern the often favorable and again unfavorable influences of the climacteric on psychoses in different individuals, are not known.

Freund reported a case of ovarian hysteria in which the removal of a dermoid tumor of the left ovary had no effect on the disease.

Olshausen agreed fully with the views of Schroeder. The small cystic ovary, while it may be associated with sexual excitement, is doubtless often a non-pathological appearance to be observed especially in pregnancy and the puerperium. He wished to observe that Hegar had made an important concession when he admitted that clinical evidence of the relation of neuroses to the function of the ovary sufficiently indicated castration.

Landau accepted the conclusions of Schroeder, that at present it is clearly empirical whether in severe neuroses we should castrate or not. Theoretical considerations do not help here. We dare recommend castration in doubtful cases when all other means have been exhausted. A criterion is furnished by one symtom which in Germany is not sufficiently acknowledged—the so-called ovarialgia. Since the report of cases made by him and Remak, he has had three cases in which castration did not relieve the pain. In these cases the ovary was free from change, and the pain was dependent on some central cause similar to that in trigeminal neuralgia. Landau thinks that in severe neuroses castration should not be done where this symptom is present. Dr. Battey reports 20 cases of oöphoralgia of which 13 were cured, 3 improved and 4 unimproved. One should also be chary of the importance given to cases reported as cured, as the same results have been reported from clitoridectomy and even cauterization of the clitoris.

Gusserow agrees with Shroeder and Olshausen. Observations like Freund's are of little importance, as it has long been known that tumors may occur in any epileptic individual, and their removal have no effect on the disease. So little is known of the essential nature of epilepsy, and its numerous causes, that, in all of these cases, before operating we should be sure that it has a relation to the ovarian function.

A brief analysis of the foregoing discussions reveals the present estimate of castration. Sir Spencer Wells regards the operation as absolutely unjustifiable in all cases where it is undertaken for the relief of nervous diseases. Hegar insists on a pathological change in some portion of the genital tract that bears an ætiological relation to the nervous disturbance. He is, however, prepared to admit that clinical evidence of the relation of the ovarian function to certain nervous diseases, *may* indicate castration, even in a normal condition of the ovary. To this latter view are committed Schroeder, Olshausen, Landau and Gusserow.

